# Positive Reinforcement Mediated by Midbrain Dopamine Neurons Requires D1 and D2 Receptor Activation in the Nucleus Accumbens

**DOI:** 10.1371/journal.pone.0094771

**Published:** 2014-04-14

**Authors:** Elizabeth E. Steinberg, Josiah R. Boivin, Benjamin T. Saunders, Ilana B. Witten, Karl Deisseroth, Patricia H. Janak

**Affiliations:** 1 Ernest Gallo Clinic and Research Center, Department of Neurology, University of California at San Francisco, San Francisco, California, United States of America; 2 Graduate Program in Neuroscience, University of California at San Francisco, San Francisco, California, United States of America; 3 Wheeler Center for the Neurobiology of Addiction, University of California at San Francisco, San Francisco, California, United States of America; 4 Princeton Neuroscience Institute and Department of Psychology, Princeton University, Princeton, New Jersey, United States of America; 5 Department of Bioengineering, Department of Psychiatry and Behavioral Sciences, Howard Hughes Medical Institute, and CNC Program, Stanford University, Stanford, California, United States of America; Roma Tre University, Italy

## Abstract

The neural basis of positive reinforcement is often studied in the laboratory using intracranial self-stimulation (ICSS), a simple behavioral model in which subjects perform an action in order to obtain exogenous stimulation of a specific brain area. Recently we showed that activation of ventral tegmental area (VTA) dopamine neurons supports ICSS behavior, consistent with proposed roles of this neural population in reinforcement learning. However, VTA dopamine neurons make connections with diverse brain regions, and the specific efferent target(s) that mediate the ability of dopamine neuron activation to support ICSS have not been definitively demonstrated. Here, we examine in transgenic rats whether dopamine neuron-specific ICSS relies on the connection between the VTA and the nucleus accumbens (NAc), a brain region also implicated in positive reinforcement. We find that optogenetic activation of dopaminergic terminals innervating the NAc is sufficient to drive ICSS, and that ICSS driven by optical activation of dopamine neuron somata in the VTA is significantly attenuated by intra-NAc injections of D1 or D2 receptor antagonists. These data demonstrate that the NAc is a critical efferent target sustaining dopamine neuron-specific ICSS, identify receptor subtypes through which dopamine acts to promote this behavior, and ultimately help to refine our understanding of the neural circuitry mediating positive reinforcement.

## Introduction

Actions that lead to beneficial outcomes are more likely to be repeated than those that do not. This process, whereby the probability of a behavioral response increases as a consequence of the outcome of that response, is referred to as positive reinforcement. ICSS is a simple behavioral model that distills positive reinforcement to its minimum neural elements. In ICSS paradigms, subjects make instrumental responses in order to deliver stimulation to a specific brain area. Sites containing dopamine neurons or their ascending projections are particularly effective in eliciting this behavior [1], and systemic administration of dopamine antagonists causes dramatic reductions in ICSS [2], strongly implicating dopamine neurons as a neural substrate. A recent study used genetically-targeted channelrhodopsin-2 (ChR2) to specifically activate VTA dopamine neurons and confirmed that dopamine neurons are indeed sufficient to drive vigorous ICSS [3], consistent with a rich literature demonstrating that VTA dopamine neurons play critical roles in learned appetitive behaviors [4], [5].

Importantly, VTA dopamine neurons send projections to many brain areas, and the specific efferent targets that support ICSS driven by optogenetic activation of dopamine neurons have not been demonstrated. Prior efforts to establish efferent targets that mediate ICSS employed electrical stimulation to reinforce operant responding [6]–[9]; however, this technique is not suitable to selectively activate a genetically-defined neural population that is intermixed with other cell types [10] or to selectively activate axon terminals innervating a single projection target. Thus, the efferent targets that mediate dopamine neuron-specific ICSS are unknown. A primary region of interest is the NAc, which is densely innervated by VTA dopamine neurons. Dopamine acting in the NAc has been extensively implicated in instrumental learning and performance for both food and drug rewards, although the exact nature of this involvement remains a matter of debate [11]–[13]. Dopamine exerts its actions in the NAc via D1 type and D2 type receptors (D1Rs and D2Rs). The relationship between striatal dopamine release, receptor activation and behavior is complex. Substantial evidence indicates that D1Rs and D2Rs engage opposing intracellular pathways [14], yet in some cases these receptors can have synergistic effects at the cellular level [15]. At the behavioral level, pharmacological studies reveal that D1Rs and D2Rs can act independently or in concert in subjects engaged in motivated behaviors [16]–[19], and selective optogenetic activation of D1R- and D2R-expressing striatal neurons can produce opposing behavioral effects [20]–[23]. The roles of D1Rs and D2Rs in supporting dopamine neuron-driven ICSS is unknown.

We sought to determine whether VTA dopamine neuron-driven ICSS was mediated by the NAc and, if so, which dopamine receptors were involved. We relied on two complementary experimental approaches to address these questions. First, we optogenetically activated VTA dopamine neuron axon terminals innervating the NAc to determine if selective activation of this pathway would support ICSS. Next, we used targeted infusions of dopamine receptor antagonists into the NAc during ICSS behavior driven by optogenetic activation of dopaminergic somata in the VTA. We found that activation of the VTA dopamine neuron projection to the NAc was sufficient to support ICSS, and that ICSS behavior mediated by VTA dopamine neurons was significantly reduced by antagonism of either D1 or D2Rs in the NAc. Taken together, these results add to a growing body of evidence implicating dopaminergic transmission in the NAc as an important element of the neural circuitry mediating positive reinforcement.

## Materials and Methods

### Experimental subjects

29 male transgenic rats (on a Long-Evans background) were used in these studies. These rats expressed Cre recombinase under the control of the tyrosine hydroxylase promoter (*Th::Cre*+, n = 19), allowing for selective targeting of dopamine neurons as described previously [3]. Their wild-type littermates (*Th::Cre*-, n = 10) were used as controls. All rats weighed >300 g at the time of surgery and were individually housed with free access to food and water. Animal care and all experimental procedures were in accordance with guidelines from the National Institutes of Health and approved in advance by the Gallo Center Institutional Animal Care and Use Committee. Surgical procedures were conducted under isoflurane anesthesia and all necessary precautions were taken to minimize animal suffering.

### Experiment 1 - surgical procedures

Standard stereotaxic surgical procedures were used to unilaterally infuse Cre-dependent virus (AAV5 Ef1α-DIO-ChR2-eYFP, titer 1.5–4×10^12^ particles/mL, University of North Carolina viral vector core) and implant optical fibers. A total of 4 µL of virus was infused into the VTA at AP −5.4 and 6.2, ML ±0.7; at each AP site 1 µL virus was delivered (0.1 µL/min) at both DV −8.4 and −7.4. The infuser was left in place for an additional 10 min to allow for diffusion. An optical fiber (Thorlabs, 300 µm diameter, 0.37 numerical aperture) was chronically implanted dorsal to the NAc (AP +1.6, ML ±1.4, DV −6.5) ipsilateral to the virus infusion. All coordinates are in mm relative to bregma and skull surface.

### Experiment 1 - behavioral procedures

120 min behavioral sessions were conducted >4 weeks post-surgery in conditioning chambers (Med Associates Inc.) contained within sound-attenuating cubicles. Session start was indicated to the rat by the illumination of a chamber light and the onset of low-volume white noise (65 dB) to mask external sounds. Two nosepoke ports, designated “active” and “inactive”, were positioned on the left chamber wall; each had three LED lights at the rear. A response at the active nosepoke port resulted in optical stimulation (20 pulses, 5 ms duration, 20 Hz, 473 nm) on a fixed-ratio 1 schedule, with the exception that a new stimulation train could not be earned until any ongoing train had finished. The LED lights in the recess of the active port were illuminated concurrent with stimulation. We chose to include a response-contingent cue in our experimental design because such cues have been show to facilitate robust operant responding over long periods in drug self-administration studies [24]. Responses at the inactive nosepoke port were recorded but had no consequence. During the first training session, both nosepoke ports were baited with a crushed cereal treat to facilitate initial investigation.

### Experiment 2 - surgical procedures

Subjects in experiment 2 received surgery as described above except that the optical fiber was targeted dorsal to the VTA (AP −5.8, ML ±0.7, DV −7.5) instead of the NAc. Additionally, 26 gauge cannulae (Plastics1) were implanted bilaterally dorsal to the NAc (AP +1.6, ML ±1.4, DV −4.8). Infusers extended 2 mm past the end of the guide cannula, for a final DV of −6.8.

### Experiment 2 - behavioral procedures

Subjects in experiment 2 underwent ICSS training as described above except that instead of single daily 120 min sessions, subjects were given a 60 min baseline session, removed from the chamber for drug infusions, and then returned for a further 60 min test session to assess drug effects on behavior. This was done because while ICSS responding was stable within a single day, the absolute magnitude of behavior emitted was variable across multiple days, even with extended training.

### Experiment 2 - drug infusions

Subjects in experiment 2 received targeted intracranial drug infusions into the NAc once ICSS behavior was established (at least 4 training sessions prior to drug administration). The following drugs were used: (1) flupenthixol, a non-selective dopamine receptor antagonist dissolved in water (10 µg; F114, Sigma); (2) SCH23390, a D1R-selective antagonist dissolved in saline (1 µg; D054, Sigma); (3) Raclopride, a D2R-selective antagonist dissolved in saline (1 µg; R121, Sigma) or (4) saline control. Drug doses were chosen based on studies that have previously demonstrated a lack of non-specific locomotor impairments [17], [18], [25]. All drug infusions were unilateral, delivered ipsilateral or contralateral to the hemisphere where VTA dopamine neurons were optogenetically stimulated, with the exception of control saline infusions which were bilateral. Doses indicate the amount delivered per hemisphere in 0.5 µL. All solutions were infused at a rate of 0.25 µL/min via 33 gauge infusers inserted into the guide cannulae; infusers were left in place for an additional 2 minutes to allow for drug diffusion. Subjects were then placed in their home cages for 10 minutes to allow the drugs to take effect before being returned to the behavioral chambers for testing. All subjects experienced all 7 treatments. The order of drugs was randomized and drug infusion testing days were preceded and followed by at least one recovery session where no treatment was given, a procedure that other groups have employed with these drugs and concentrations [18], [25].

### Experiments 1 and 2 – optical stimulation methods

Prior to all behavioral sessions, rats were gently attached to custom-made optical cables (200 µm diameter, 0.37 numerical aperture) encased in durable metal covering (Penflex, SL-SS-001). The cables were secured to the rat's implanted optical fiber with a ceramic sleeve (Fiber Instrument Sales) and attached at the other end to an optical commutator (Doric Lenses). The commutator was mounted on a counterbalanced lever arm to facilitate unhindered behavioral responding, and connected via a second cable to a 100–150 mW DPSS laser (OEM Laser Systems). Light output during individual light pulses was estimated to be ∼2 mW at the tip of the intracranial fiber. This value was derived by measuring the average light power when the laser was pulsed at the parameters used for our experiments (20 Hz, 5 ms pulse width) and then correcting for the duty cycle (in this case, dividing by 0.1). Based on this value we estimate that light power density at the tip of the fiber was ∼7 mW/mm^2^ (calculated using www.optogenetics.org/calc). Light power was measured before and after every behavioral session to ensure that all equipment was functioning properly.

### Data analysis and statistics

In experiment 1, the total number of active and inactive nosepoke responses made across multiple training days was compared within and between *Th::Cre*+ and *Th::Cre*- groups. In experiment 2, the effect of drug infusions on active nosepoke responding was assessed by expressing post-drug responding as a percentage of a pre-drug baseline value, and the numbers of c-Fos+ cells were compared between *Th::Cre*+ and *Th::Cre*- groups. Parametric (one- or two-way ANOVA followed by post-hoc Student-Newman-Keuls tests) or non-parametric (Wilcoxon signed-rank tests with Bonferroni corrections; Mann-Whitney rank sum test, Friedman repeated-measures ANOVA) tests were used where appropriate.

### Histology

Immunohistochemical detection of TH and YFP was performed in all subjects used in Experiments 1 and 2 as described previously [3]. C-Fos immunohistochemistry was performed in a separate cohort of rats that received prior ICSS training. Following a 2-hour ICSS session, rats were deeply anesthetized with sodium pentobarbital and perfused transcardially with 0.9% saline followed by 4% paraformaldehyde. After removal, brains were cryoprotected in 25% sucrose for >48 hours and sectioned coronally at 50 µm on a freezing microtome. Free-floating sections were washed sequentially with (1) phosphate buffered water (PB; pH 7.4), (2) 50% EtOH, (3) 50% EtOH with 0.009% hydrogen peroxide and (4) 5% donkey serum, all for 30 min. Sections were then incubated in a primary antibody (goat anti-c-Fos; 1∶1000, Santa Cruz) solution containing 0.2% triton and 2% donkey serum for 48 hours at 4°C. After several washes with PB, a secondary antibody (1∶200, biotinylated donkey anti-goat, Jackson ImmunoResearch) solution that contained 0.2% triton and 2% donkey serum was applied overnight at 25°C. After further PB washes, sections were incubated with ExtrAvidin (1∶2500, Sigma) for 2 hours at 25°C. After additional washes, sections were transferred to a diaminobenzidine solution for 5.5 minutes. The total number of c-Fos+ cells were counted within the borders of the NAc (n = 8 sections per rat) and VTA (n = 4 sections per rat) by an observer who was blinded to both the animal's genotype and the hemisphere where the optical fiber had been implanted. Hemisphere-blinding was only possible for counts in the NAc, as the optical fiber itself was clearly visible in VTA sections. Fluorescent triple-labeling for YFP, TH and c-Fos was conducted in a subset of animals from Experiment 2 that were sacrificed immediately after a 2-hour ICSS session. Sources for antibodies were as follows. Primary: mouse anti-GFP (1∶1500, Invitrogen) rabbit anti-TH (1∶1500, Fisher Scientific), and goat anti-Fos (1∶500, Santa Cruz). Secondary: Alexa Fluor 488 or 594 dyes (1∶200, Invitrogen) or CF633 (1∶200, Biotium Inc.) Although optical fiber placements and virus expression varied slightly between subjects, none were excluded based on histology.

For the quantification of ChR2-YFP expression (measured as fluorescence intensity) in experiment 1, VTA sections from *Th::Cre*+ rats were imaged using identical magnification and exposure settings on a confocal microscope. The portion of the image containing the VTA was manually isolated as a region of interest and fluorescence intensity was calculated in this area using imageJ software.

## Results

### Experiment 1

**Figure 1:**
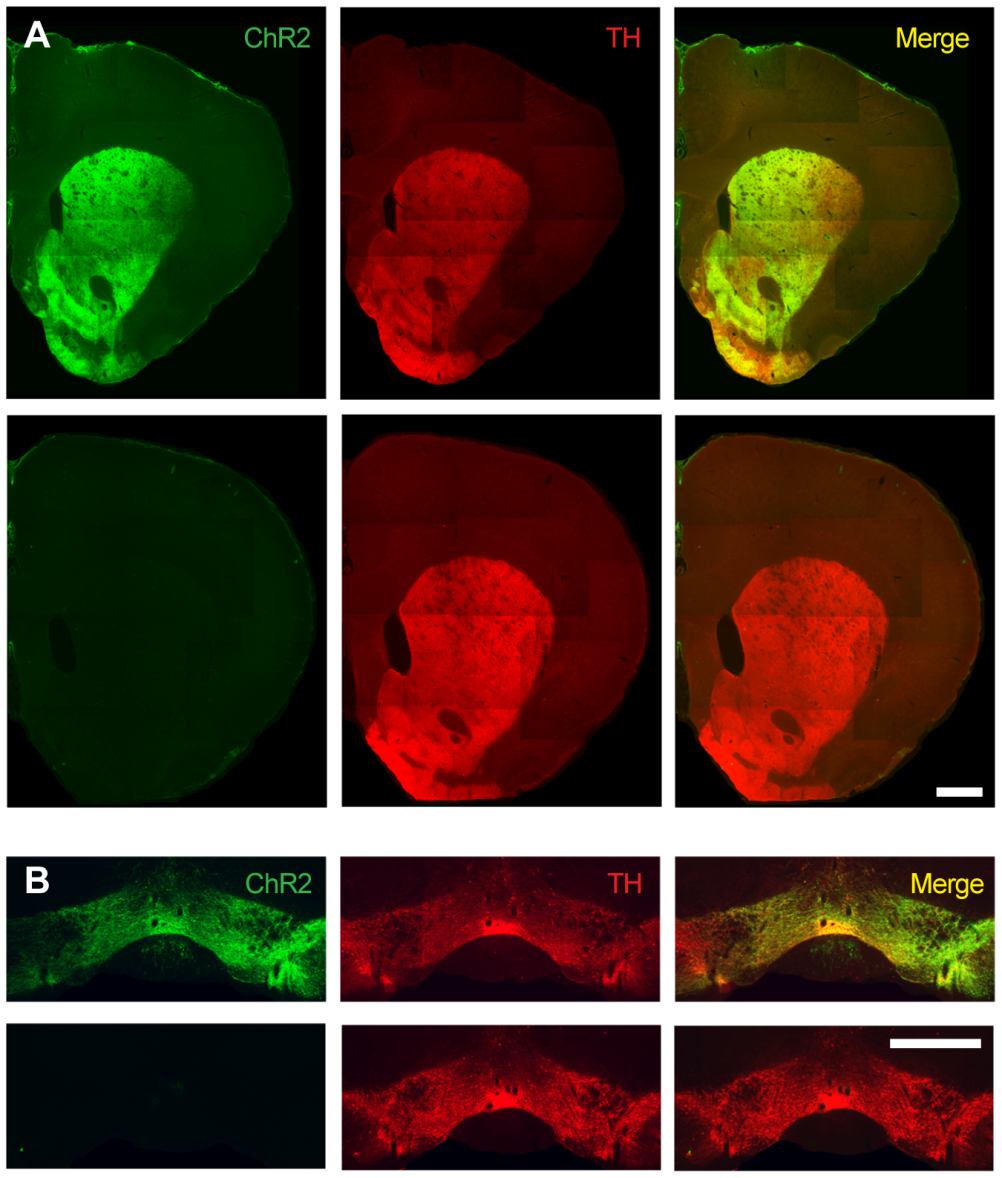
*ChR2-YFP expression is limited to Th::Cre+ rats*. (A) ChR2-YFP and TH staining in the striatum of representative *Th::Cre*+ (top) and *Th::Cre*- (bottom) rats. Both rats received identical virus injections targeted to the VTA. (B) ChR2-YFP and TH staining in the midbrain of representative *Th::Cre*+ (top) and *Th::Cre-* (bottom) rats. Both rats received identical virus injections targeted to the VTA. In A-B, scale bar  = 1 mm.

**Figure 2:**
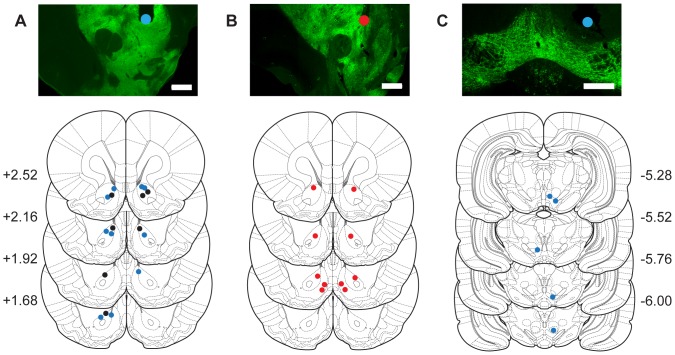
*Example and group histology*. (A) Top; representative striatal ChR2-eYFP expression and NAc optical fiber placement for experiment 1 (blue dot indicates fiber tip). Bottom; histological reconstruction of optical fiber tip placements for subjects receiving intra-NAc optical stimulation. Blue dots indicate tip placement in *Th::Cre+* rats; black dots indicate tip placement in *Th::Cre-* rats. (B) Top; representative striatal ChR2-eYFP expression and NAc infuser tip placement for experiment 2 (red dot indicates infuser tip). Bottom; histological reconstruction of infuser tip placements for subjects receiving intra-NAc drug infusions. (C) Top; representative VTA ChR2-eYFP expression and optical fiber placement for experiment 2 (blue dot indicates fiber tip). Bottom; histological reconstruction of optical fiber tip placements for subjects receiving intra-VTA optical stimulation. In A-C scale bars  = 500 µm.

**Figure 3:**
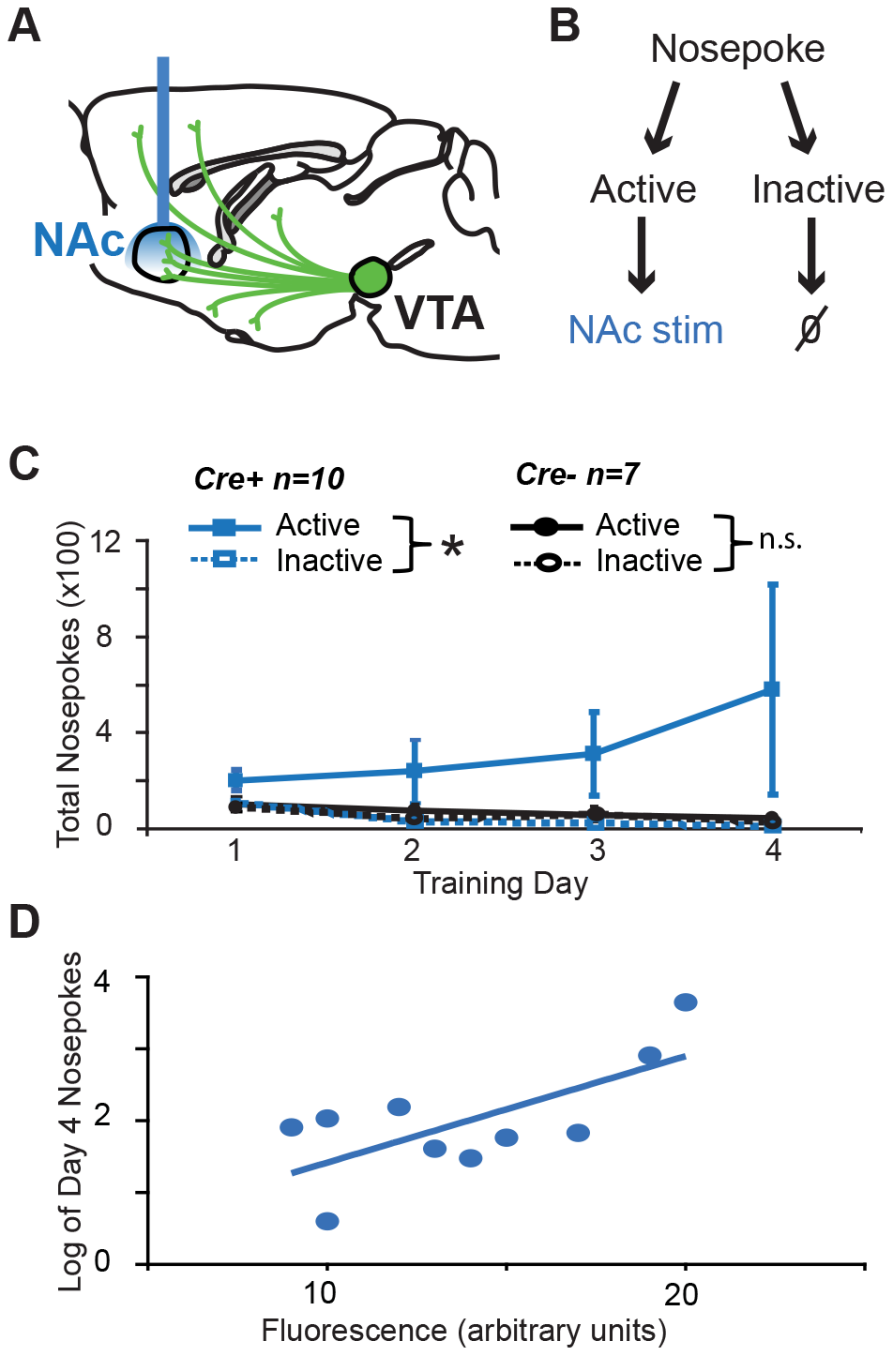
*Optical stimulation of VTA dopamine efferents to NAc supports self-stimulation.* (A) Virus was infused into the VTA, and an optical fiber was implanted targeting the NAc. (B) Schematic of ICSS task. A response at the active nosepoke port was reinforced with optical stimulation (20 pulses, 20 Hz, 5 ms duration, 473 nm) on an FR1 schedule. Responses at the inactive nosepoke port had no consequence. (C) Active and inactive nosepoke responding for *Th::Cre+* and *Th::Cre-* rats during 4 days of FR1 training (120 min sessions). *Th::Cre+* rats performed significantly more active than inactive nosepokes on all 4 days (2-tailed Wilcoxon Rank test with Bonferroni correction, *p<0.05) (D) YFP fluorescence in the VTA of *Th::Cre+* rats correlates with the log of FR1 response rate on training day 4 (p = .026; r^2^ = 0.482).

We initially set out to determine whether selective activation of dopaminergic axon terminals innervating the NAc would be sufficient to support ICSS. We performed our experiments in a recently developed transgenic rat line where Cre recombinase expression is driven by the tyrosine hydroxylase (*Th*) promoter (*Th::Cre* rats) in order to gain selective control over dopamine neuron activity [3]. Subjects received intra-VTA injections of a Cre-dependent virus encoding ChR2; ChR2 expression was restricted to TH+ neurons and their efferent projections in *Th::Cre*+ rats (Fig. 1A,B). After virus injection, *Th::Cre*+ rats or their wild-type (*Th::Cre*-) littermates were chronically implanted with an optical fiber targeting the NAc (Fig. 2A, 3A). 6–8 weeks later, all subjects were given ICSS training sessions. During ICSS training, each response at an active nosepoke port resulted in a 1-second (20 pulses, 5 ms duration, 20 Hz) optical stimulation train delivered intracranially to the NAc, parameters that we have previously established elicit time-locked spiking in VTA DA neurons in *in vitro* and anesthetized *in vivo* preparations, as well as robust dopamine release in NAc brain slices [3]. LED lights in the recess of the active port were illuminated concurrent with the optical stimulation train. Responses at an otherwise identical inactive nosepoke port had no consequence (Fig. 3B). *Th::Cre*+ rats made more active than inactive nosepoke responses on all 4 training days (Fig. 3C, 2-tailed Wilcoxon rank test with Bonferroni correction; days 1–4 p = 0.016, 0.016, 0.04 and 0.008 respectively), while *Th::Cre*- rats did not (Fig. 3C, 2-tailed Wilcoxon Rank test with Bonferroni correction; days 1–4 p = 1.0, 0.064, 1.0 and 0.876 respectively). A comparison of active nosepoke responding between *Th::Cre*+ and *Th::Cre*- groups failed to reach significance (2-tailed Mann-Whitney test, p = 0.107 on day 4); however, variability in virus expression may account for the lack of a between-group effect. In support of this, ChR2 expression strength in the VTA of *Th::Cre*+ rats was significantly correlated with total responses made at the active port (Fig. 3D; p = 0.026, r^2^ = 0.482), and the *Th::Cre*+ rats that displayed above-average expression of ChR2 (n = 4) performed significantly more active nosepokes than *Th::Cre*- rats on day 4 (p = 0.024; 2-tailed Mann-Whitney test). Thus, optical activation of the dopaminergic projection to the NAc is sufficient to support ICSS, confirming an important role for this pathway in the neural basis of positive reinforcement.

### Experiment 2

**Figure 4:**
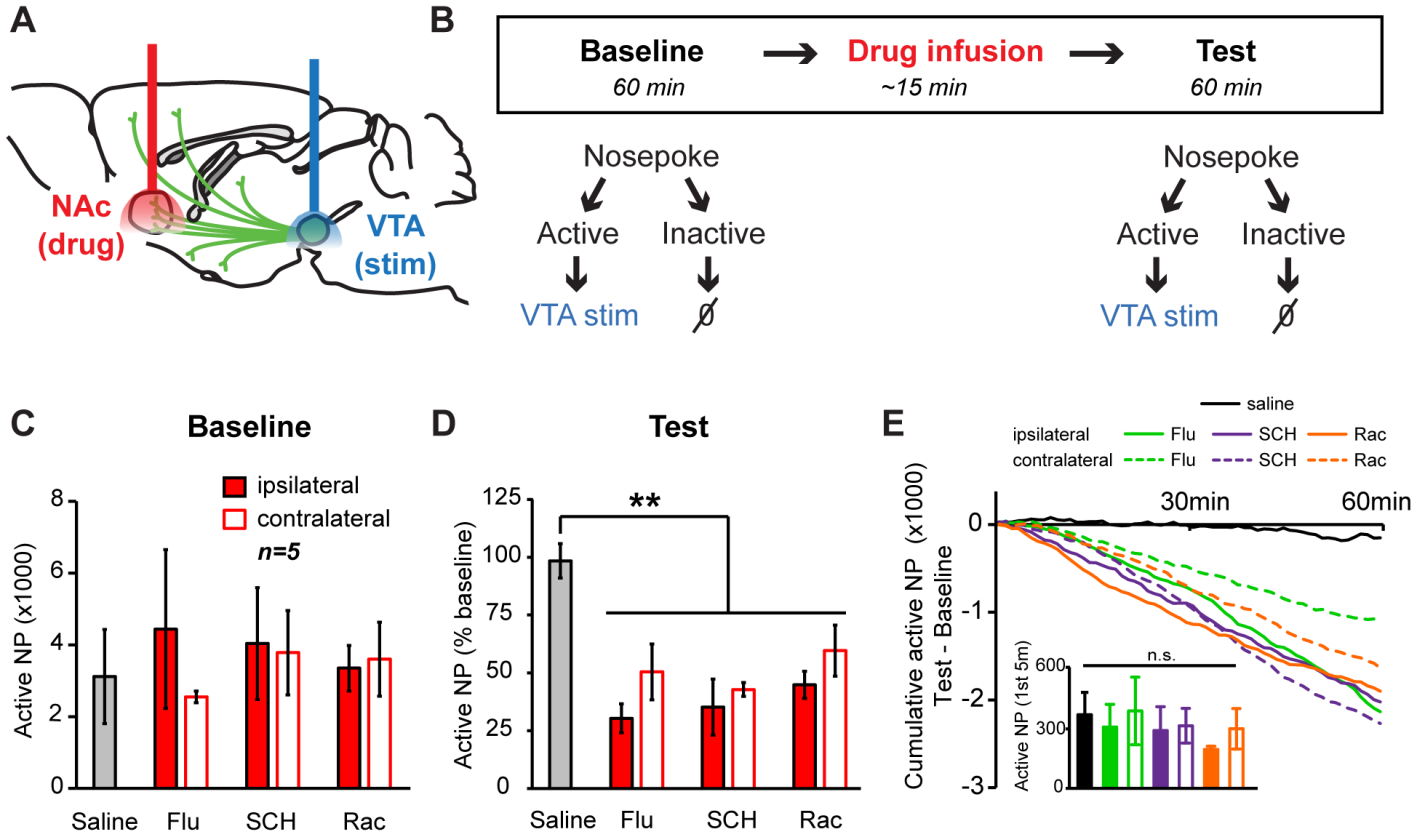
*Self-stimulation driven by VTA dopamine neurons is attenuated by intra-NAc D1 and D2 receptor antagonists.* (A) Virus was injected into the VTA and an optical fiber was targeted to this region; cannulae were targeted to the NAc. (B) Schematic of ICSS task with drug infusions. A 60 min baseline ICSS session was administered where responses at the active nosepoke port were reinforced with optical stimulation (20 pulses, 20 Hz, 5 ms duration, 473 nm) on an FR1 schedule. After intra-NAc drug infusion, a 60 min test ICSS session was administered that was identical to the baseline session. (C) Active nosepoke responding during baseline (pre-drug) sessions. There were no differences in responding (Friedman one-way repeated measures ANOVA, main effect of treatment χ^2^(6) = 6.771, p = 0.343) (D) Active nosepokes during test (post-drug) sessions quantified as a percentage of baseline responding. Relative to saline, all drug treatments significantly reduced responding (one-way repeated measures ANOVA, main effect of treatment *p*<0.001, **post-hoc test vs. saline *p*<0.01). (E) Cumulative active nosepokes made during the 60 min test session, with the corresponding value from baseline sessions subtracted to highlight differential responding. Note that responding from saline sessions remains close to the baseline value while responding after drug treatment steadily decreases. Data represent the mean of all rats (n = 5), SEM not shown. Inset, total number of active nosepokes made in the first 5 minutes of each test session without baseline subtraction. There were no differences in this measure (Friedman one-way repeated measures ANOVA, main effect of treatment χ^2^(6) = 5.829, p = 0.443).

Next, we combined our optogenetic approach with pharmacological tools that allowed us to assess the contribution of dopamine acting on specific dopamine receptor subtypes to ICSS behavior. *Th::Cre*+ rats were injected with Cre-dependent ChR2 virus unilaterally into the VTA, and an optical fiber was implanted dorsal to this structure (Fig. 2C, 4A). Additionally, bilateral cannulae were implanted targeting the NAc (Fig. 2B, 4A). After a recovery period, subjects were initially allowed to acquire ICSS behavior where each response at the active nosepoke resulted in a 1-second (20 pulses, 5 ms duration, 20 Hz) optical stimulation train delivered intracranially to dopamine somata in the VTA, concurrent with illumination of the LED lights in the recess of the active port. Once robust ICSS behavior had been established (at least 4 training sessions, mean ± SEM 2939.9±1584.6 active and 5.3±3.2 inactive nosepokes per hour) subjects received test sessions where dopamine receptor antagonists were infused into the NAc prior to ICSS training. We used a within-session, within-subject experimental design. Subjects were allowed to respond for dopamine-neuron ICSS during a 1-hour baseline session. Then, dopamine antagonists were infused into the NAc unilaterally (either ipsilateral or contralateral to the optical fiber implanted above the VTA), and subjects were returned to the behavioral chambers where they received an additional 1-hour ICSS test session (Fig. 4B). Drug effects were assessed by comparing post-drug active nosepoke responding to the same subject's pre-drug baseline value. All subjects maintained robust ICSS behavior during baseline sessions *prior* to drug infusion (Friedman one-way repeated measures ANOVA, main effect of treatment χ^2^(6) = 6.771, p = 0.343, Fig. 4C). We found that administration of dopamine antagonists into the NAc significantly reduced ICSS behavior, expressed as a percentage of pre-drug baseline responding, during test sessions (one-way repeated measures ANOVA, main effect of treatment F_6,34_ = 6.414, p<0.001, Fig. 4D). Planned post-hoc comparisons revealed that unilateral infusions of flupenthixol (a non-selective dopamine antagonist), SCH23390 (a D1R-specific antagonist) or raclopride (a D2R-specific antagonist) dramatically decreased ICSS behavior as compared to saline vehicle (all p's vs. saline <0.007). Decreased ICSS behavior observed in drug-treated rats was unlikely to have resulted from motor impairments, as active nosepoke responding was similar under all treatment conditions during the first 5 minutes of the test session (Friedman one-way repeated measures ANOVA, main effect of treatment χ^2^(6) = 5.829, p = 0.443; Fig. 4E, inset).

Interestingly, subsequent analyses demonstrated that ipsilateral or contralateral dopamine antagonist infusions (respective to the optical fiber) caused similar decreases in ICSS behavior (2-tailed Wilcoxon rank test with Bonferroni correction, all p's>0.564). This finding was surprising, since the dopaminergic projection from the VTA to the NAc is thought to be almost exclusively unilateral [26]. We hypothesized that the effects of contralateral drug infusions were a consequence of optical activation of VTA dopamine neurons and their projections to the NAc in the contralateral hemisphere during ICSS. This hypothesis is supported by the observation that our unilateral virus injections resulted in bilateral ChR2 expression in VTA neurons (Fig. 1B, 2C), likely because of the VTA's midline location and the large volume of virus we infused to ensure robust infection. Recent efforts to quantify the propagation of light in living neural tissue (using optical fibers with properties similar to those used in the present experiments) demonstrate that the width of light spread in intact brain is quantitatively similar to its depth [27], indicating that light may have reached ChR2-expressing dopamine neurons in the contralateral VTA and evoked dopamine release in the corresponding NAc.

**Figure 5:**
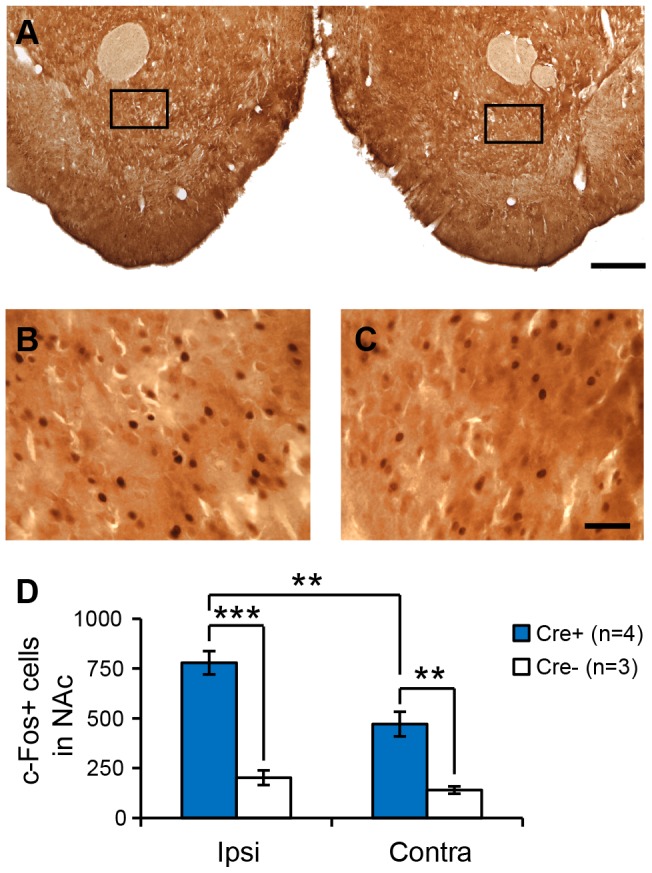
*ICSS elicits bilateral c-Fos expression in NAc neurons*. (A) C-Fos immunohistochemical staining in the NAc of a *Th::Cre+* rat sacrificed immediately after an ICSS session. Black boxes correspond to areas magnified in B and C. (B) High-magnification view showing c-Fos+ cells both ipsilateral and contralateral (C) to the optical fiber. (D) Quantification of c-Fos+ cells in the NAc (n = 8 sections per rat, n = 7 rats). There were more c-Fos+ cells in *Th::Cre*+ rats in both hemispheres of the NAc as compared to *Th::Cre*- controls (***p<0.001 ipsi, **p<0.01 contra, Student-Newman-Keuls post-hoc test). *Th::Cre*+ rats also had stronger c-Fos expression in the ipsilateral as compared to contralateral hemisphere (**p<0.01, Student-Newman-Keuls post-hoc test) Ipsi/contra designation refers to the location of the optical fiber in the VTA. Scale bar  = 500 µm in A and 50 µm in B–C.

**Figure 6:**
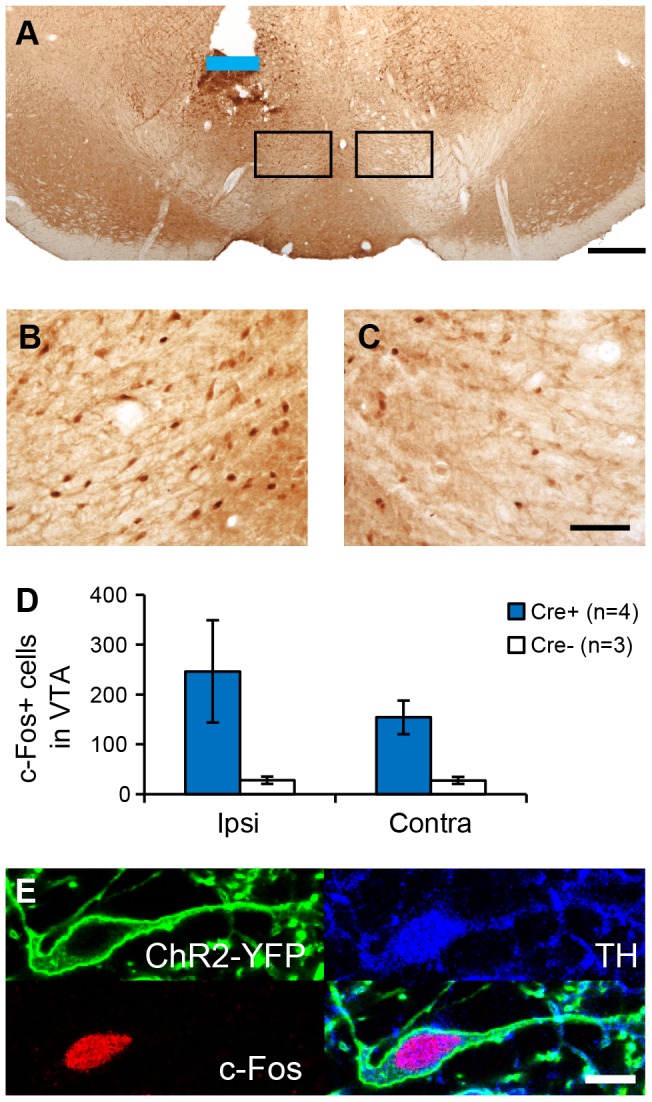
*C-Fos expression in VTA neurons after ICSS*. (A) C-Fos immunohistochemical staining and optical fiber placement in the VTA of a *Th::Cre+* rat sacrificed immediately after an ICSS session. Blue line indicates location of optical fiber tip. Black boxes correspond to areas magnified in B and C. (B) High-magnification view of c-Fos staining showing c-Fos+ cells both ipsilateral and contralateral (C) to the optical fiber. (D) Quantification of c-Fos+ cells in the VTA (n = 4 sections per rat, n = 7 rats). Although there was a trend for greater c-Fos expression in *Th::Cre*+ rats (two-way repeated measures ANOVA, main effect of genotype p = 0.083), no comparison reached statistical significance. (E) High-magnification view of ChR2-eYFP and TH immunohistochemical staining in a c-Fos+ neuron showing colocalization of all three proteins. Scale bar  = 500 µm in A; 50 µm in B–C; and 10 µm in E.

We used immunohistochemical detection of c-Fos, a marker commonly used to identify recently active neurons, in order to determine if contralateral NAc and/or VTA neurons were activated during ICSS behavior. Subjects were sacrificed immediately after a 2-hour ICSS session wherein *Th::Cre*+ rats (n = 4) and *Th::Cre*- rats (n = 3) performed a mean ± SEM of 8063±151 and 6±4 active nosepokes, respectively; the number of c-Fos+ cells in the NAc and VTA was counted by an experimenter blind to the subject's genotype. We observed significantly more c-Fos+ cells in the NAc of *Th::Cre*+ rats as compared to *Th::Cre*- controls (two-way repeated measures ANOVA, main effect of genotype F_1,13_ = 54.262, p<0.001, Fig. 5). C-Fos was elevated in both hemispheres in *Th::Cre*+ rats, (*Th::Cre*+ vs. *Th::Cre*- p<0.001 within ipsi, p = 0.002 within contra, Student-Newman-Keuls post-hoc tests), although overall c-Fos expression was higher ipsilaterally in *Th::Cre*+ rats (two-way repeated measures ANOVA, hemisphere x genotype interaction F_1,13_ = 7.817, p = 0.038, ipsi vs. contra within *Th::Cre*+ p = 0.003 Student-Newman-Keuls post-hoc test). In the VTA, we observed a trend towards increased c-Fos expression in *Th::Cre*+ rats (two-way ANOVA, main effect of genotype F_1,13_ = 4.659, p = 0.083, Fig. 6), but no indication of inter-hemispheric differences (main effect of hemisphere, F_1,13_ = 1.187, p = 0.326, hemisphere x genotype interaction F_1,13_ = 1.17, p = 0.329). C-Fos+ cells in the VTA often co-expressed TH and ChR2-YFP (Fig. 6E), indicating that these cells are likely to be light-activated dopamine neurons. These data demonstrate that our unilateral optical manipulation caused bilateral activation of neurons within the NAc, suggesting that both ipsilateral and contralateral drug infusions in this structure are likely to disrupt behavior, in accord with our findings.

## Discussion

Our data demonstrate that the dopaminergic projection to the NAc causally contributes to positive reinforcement. Using Cre-dependent opsin expression in transgenic rats, we were able to manipulate dopamine neuron activity with genetic, anatomical and temporal precision in behaving subjects engaged in ICSS. We found that selective activation of dopaminergic terminals innervating the NAc was sufficient to reinforce acquisition of an instrumental response, demonstrating a causal relationship between activation of this neural pathway and behavior. In addition, we found that ICSS behavior driven by optical activation of dopamine somata in the VTA was significantly attenuated by localized infusion of dopamine antagonists into the NAc, further implicating this pathway in positive reinforcement. By examining c-Fos expression elicited by ICSS, we determined that our unilateral optical manipulation resulted in bilateral neural activation within the NAc. Consistent with this observation, dopamine receptor antagonist infusions into either hemisphere induced behavioral changes. Dopamine neuron-specific ICSS required activation of both D1 and D2Rs, as antagonists of either receptor significantly reduced ICSS behavior.

An interesting feature of our data is the order-of-magnitude difference in ICSS behavior evoked by stimulation of dopamine neuron somata in the VTA (e.g., Fig. 4C, 1 hr session) and stimulation of dopaminergic axons within the NAc (e.g., Fig. 3C, 2 hr session). This could be due to anatomical differences in the density of dopamine neurons/axons within the area of illumination or, alternatively or in addition, may indicate that VTA dopamine neurons also support reinforcement via connections with other brain regions. However, the substantial reductions in somata-driven ICSS behavior induced by intra-NAc dopamine antagonist infusions (which presumably impact a larger volume of tissue than optical activation of dopaminergic axon terminals in the NAc) suggest that limited light penetration within a large structure is a likely, if partial, explanation for the discrepancy. It is worth noting that even after unilateral dopamine antagonist infusions into the NAc, operant behavior was substantially reduced (30–60% of baseline) but not entirely eliminated. This residual responding could be mediated by a variety of neural substrates, including dopaminergic projections to the non-infused side of the NAc, incomplete drug spread within the targeted NAc, other neurotransmitters besides dopamine acting in the NAc, or projections from dopamine neurons within the VTA to efferent targets other than the NAc.

While ipsilateral drug infusions consistently produced numerically greater reductions in ICSS behavior than contralateral infusions (e.g. we observed 30.4±6.2% of baseline responding post-ipsilateral flupenthixol, and 50.4±12.0% post-contralateral flupenthixol), these effects were statistically indistinguishable when the data were considered collectively. This similarity in magnitude is intriguing given clear inter-hemispheric differences in ChR2 and c-Fos expression. Critically, the pharmacological actions of dopamine antagonists reported here would be expected to block all effects of dopamine, whether released by optical stimulation or endogenous neural processing. It is possible that endogenous dopamine release must be intact in both hemispheres to permit normal ICSS behavior, although this idea is not supported by prior work which has demonstrated that ICSS behavior for an electrical stimulation reinforcer is minimally affected by unilateral lesions of ascending dopaminergic projections [9]. Even so, it is interesting to speculate that ipsilateral and contralateral antagonist infusions may alter behavior through partially distinct psychological mechanisms, with ipsilateral infusions acting primarily to reduce the reinforcing properties of optical stimulation and contralateral infusions acting primarily to reduce general motivation necessary to engage in vigorous ICSS behavior.

Our results are in accord with a rich literature implicating VTA dopamine neurons, and their major efferent projection to the NAc, in reward-related behaviors [4], [5], [12], [28]. However, the present results build on previous work in important ways. Until recently, ICSS studies relied on stimulating electrodes to briefly increase neural activity. However, electrical stimulation activates a heterogeneous neural population whose spatial distribution is difficult to predict [10], [29], a significant issue in a brain region such as the VTA where non-dopamine neurons constitute a sizeable minority (∼40%; [4]). Thus, it is difficult to ascribe observed behavioral effects to dopamine neurons with certainty. Here, we used genetically-targeted tools that permitted selective and specific activation of dopamine neurons, thereby circumventing this problem. Interestingly, prior studies that used electrical stimulation of the VTA to drive ICSS found that intra-NAc antagonism of D1Rs, but not D2Rs, attenuated ICSS [19], [30]. In contrast, our results demonstrate that D1Rs and D2Rs both contribute to this behavior. It has been suggested that activation of D1 and D2 receptors by dopamine is concentration dependent, with low concentrations preferentially activating D2 receptors and high concentrations additionally recruiting D1 receptors [31], [32]. The extracellular concentration of exogenously-evoked dopamine has been shown to be highly dependent on the stimulation parameters employed [33], [34]. Thus, discrepancies in the receptor dependence of electrical and optical ICSS may be explained by differences in the concentration of dopamine they evoke in terminal fields. In our study, we used stimulation parameters that approximate the natural firing patterns of dopamine neurons in response to natural rewards and cues. The location and identity of dopamine receptors involved in ICSS mediated by other optical stimulation parameters remains an interesting subject for future exploration. D2Rs are found both pre- and post-synaptically within the NAc [35], and receptor activation at these sites can produce divergent effects. Because our pharmacological manipulations cannot distinguish between these sites of action, the cellular localization of the receptors responsible for generating the behavioral effects we observed remains to be demonstrated.

Other recent studies have also used optogenetics to examine the contributions of midbrain dopamine neurons to positive reinforcement and learning [3], [34], [36]–[39]. In agreement with our prior findings [3] and the present findings, both obtained in rats, Kim et al. (2012) and Rossi et al. (2013) observed dopamine neuron ICSS in mice. In contrast, Adamantidis et al. (2011) did not observe dopamine neuron ICSS in *Th::Cre*+ mice; it is not clear which procedural, or other, variables account for this difference. However, all of the above mentioned efforts have focused on the behavioral effects of manipulating a mixed population of dopamine neurons with diverse projection targets. In contrast, the experiments described here were designed to isolate the contribution of a specific dopaminergic projection (VTA to NAc) to behavior. Because dopamine neurons are embedded in a complex and multifunctional circuitry, such pathway-specific approaches are essential in developing a detailed understanding of the ways in which this important neural population contributes to behavior.

Midbrain dopamine neurons are known to co-release other neurotransmitters and peptides in addition to dopamine, and these molecules may be important mediators of the signals relayed by dopamine neurons to the rest of the brain [5]. Thus, pharmacological controls are required to determine whether the behavioral consequences of optogenetically activating dopamine neurons are in fact due to cellular actions of dopamine. Here, we demonstrate that ICSS driven by optical activation of VTA dopamine neurons depends on the actions of dopamine at its receptors in the NAc (Fig. 4). Our results represent an advance over previous studies [3], [34], [36]–[39] that did not include these controls. It is of interest to explore potential roles of other co-released transmitters and projections to efferent targets other than the NAc in future studies, as our results to not preclude an important function for these anatomical connections in positive reinforcement.

The present findings indicate that the VTA to NAc projection is positively reinforcing in that it can support acquisition and performance of ICSS; these studies do not determine the distinct behavioral mechanisms that may contribute to this effect. The behavioral procedure we used in the present study was designed such that each nosepoke that resulted in dopamine neuron stimulation also resulted in simultaneous presentation of a visual cue within the nosepoke operandum. Thus, it remains to be determined whether the optical stimulation reinforced the instrumental action, or via association of the stimulation with the cue, allowed the response-paired cue to act as a conditioned reinforcer. Of note, we recently showed that sucrose reward-paired dopamine neuron stimulation can promote conditioned responding to reward cues, in agreement with a role for dopamine as a reward prediction error signal in temporal difference learning (TDL) models [39], and the attribution of incentive value to a dopamine-paired cue may be mediated by such a mechanism. The acquisition of ICSS can also be explained within a TDL framework as a dopamine-mediated increase in action value (c.f., [40]). The elucidation of the learning mechanism at work in the present study awaits further experimentation.
